# Glutamine supplementation

**DOI:** 10.1186/2110-5820-1-25

**Published:** 2011-07-18

**Authors:** Jan Wernerman

**Affiliations:** 1 Department of Intensive Care Medicine, Karolinska University Hospital Huddinge, Karolinska Institutet, Stockholm, Sweden

## Abstract

Intravenous glutamine supplementation is standard care when parenteral nutrition is given for critical illness. There are data of a reduced mortality when glutamine supplementation is given. In addition, standard commercial products for parenteral nutrition do not contain any glutamine due to glutamine instability in aqueous solutions. For the majority of critical ill patients who are fed enterally, the available evidence is insufficient to recommend glutamine supplementation. Standard formulation of enteral nutrition contains some glutamine: 2-4 g/L. However, this dose is insufficient to normalize glutamine plasma concentration.

Plasma concentration of glutamine is low in many patients with critical illness and a low level is an independent risk factor for mortality. A low plasma glutamine concentration is the best indicator of glutamine depletion. Data are emerging about how the endogenous production of glutamine is regulated. We know that skeletal muscle is the major producer of glutamine and that a part of the profound depletion of skeletal muscle seen in critical illness is a reflection of the need to produce glutamine.

Glutamine is utilized in rapidly dividing cells in the splanchnic area. Quantitatively most glutamine is oxidized, but the availability of glutamine in surplus is important for the de novo synthesis of nucleotides and necessary for cell division and protein synthesis. More knowledge about the regulation of the endogenous production of glutamine is needed to outline better guidelines for glutamine supplementation in the future.

## Recommendation

Presently the recommendation from ESPEN and ASPEN/SCCM is to add intravenous (IV) glutamine supplementation when total parenteral nutrition (TPN) is given to critically ill patients [1-3]. In all guidelines, this is given a level A recommendation. This evidence is based on several meta-analyses, and the recommendations also include a dose of glutamine of 0.3-0.5 g/kg/day. This dose will normalize plasma glutamine concentration in almost all critically ill patients [4,5].

The problem arises when the patients are fed differently than by TPN. This includes enteral nutrition, combined enteral and parenteral nutrition, and hypocaloric nutrition. An insufficient dose of glutamine may be added to these states. In all these settings, the existing evidence is less conclusive.

## Route of food administration

Many colleges, investigators, and opinion-leaders feel that the route of nutrient administration makes a difference in critically ill patients. Usually the opinion is that enteral nutrition is superior to parenteral nutrition. This is probably true for infectious complications in patients randomized to enteral or parenteral nutrition, but no studies or meta-analyses demonstrate a mortality difference [6-10]. In fact the meta-analysis that demonstrates a difference, does so in favor of parenteral nutrition [10].

Numerous arguments may be given about why the design of studies that support the existing meta-analyses are not adequate: for example, only patients with a functional gastrointestinal tract were included; no glucose control; different caloric intake in groups; no dropout information; no blinding.

In the existing scoring systems for outcomes, there is no marker for gastrointestinal function. This is largely related to the absence of a suitable surrogate parameter for scoring. On the other hand, there is solid information that a successful enteral feeding, indicative of a functional gastrointestinal tract, is a predictor for a favorable outcome in patients with a similar mortality risk as estimated from the conventional scoring systems [2,11,12]. Success of enteral feeding may be enhanced by technical or pharmaceutical techniques, but presently there is very little evidence that such enhancement gives an outcome benefit even if it makes enteral nutrition more successful [13].

## Route of glutamine administration

As stated earlier, IV glutamine supplementation of 0.3-0.5 g/kg/day provides an improved outcome for patients on TPN [14-17]. This corresponds to an exogenous glutamine supplementation of 20-30 g/day, which normalizes the plasma concentration in most critcally ill patients [4,5]. Healthy subjects have an endogenous glutamine production of 50-80 g/day [18-21]. Most of the de novo glutamine synthesis takes place in skeletal muscle and is thereafter exported to the splanchnic area to be used mostly in enterocytes and immune cells [22,23]. In the critically ill, glutamine production is not altered, but the production is insufficient to keep up the plasma concentration [20,24,25].

IV glutamine supplementation results in a uniform uptake of glutamine across the splanchnic area, similar to what happens with the endogenously produced glutamine. IV administration of glutamine is well characterized and the elimination rate from plasma is fast [4]. This pharmacokinetic profile makes it preferable to use a constant infusion for the IV glutamine supplementation. This may be as an additive to the parenteral nutrition or as a separate IV infusion of glutamine. In the latter case, this may be administered in a central or peripheral vein [26]. If a commercially available 20% glutamine containing dipeptide solution is used, the osmolarity is high (approximately 900 mosm/L); however, because the pH is neutral, this does not irritate the peripheral veins.

In the case of enteral glutamine supplementation, the kinetics is much less well characterized [27]. Overall, the uptake of crystalline or dipeptide glutamine is fast and occurs in the upper part of the jejunum [18,28]. Only a fraction of the absorbed glutamine can be recovered in the portal blood. This is indicative of elimination in the gut, most likely in the enterocytes and immune cells present there. From portal blood through the liver into the systemic circulation another fraction of the absorbed glutamine is utilized in the liver. Therefore, the so-called first "pass elimination" is substantial: 40-90%. It may be argued that this uptake is adequate, that the enterocytes and the immune cells actually are the targets for the endogenous glutamine production [22]. This is true, but at the same time, the complete uptake of the enterally administered glutamine in the upper part of the jejunum leaves the remaining part of the gastrointestinal tract unsupported by the enteral route. The effect on plasma glutamine concentration of an enterally administered glutamine supplementation is marginal [29-31]. So, after enteral glutamine supply, the low plasma concentration in critically ill is still low. It is highly likely that when enteral nutrition is not possible, a supply of parenterally administered glutamine becomes critical for the intestine. This may to some extent be compensated for by other amino acids when enteral nutrition is functional.

### Level of feeding

The studies that demonstrate a favorable effect of glutamine are all studies where patients are fed according to their measured or estimated energy expenditure [14,15,17]. This is one possible explanation why studies with enterally supplemented patients on enteral nutrition have not been conclusive. For patients on parenteral nutrition, there are no reports employing a hypocaloric nutrition supplemented with glutamine.

It is well known that overfeeding critically ill patients may cause harm [2,32]. Overfeeding most often is practised when parenteral nutrition is given. Today this may be when large doses of lipid emulsion are given as a vehicle for drugs, or when enteral and parenteral nutrition is combined and the two routes of administration are not cleared between each other properly. Still if the caloric support is decided according to body weight, a considerable overfeeding may happen in individual cases. Adding glutamine supplementation may further boost the overfeeding. Measuring the energy expenditure by indirect calorimetry decreases the risk of overfeeding and enhances the quality of care in the ICU [33,34].

### Plasma concentration

The fact that glutamine deficiency defined as a low plasma concentration at ICU admittance is an independent mortality predictor [35,36] makes it mandatory that future studies of exogenous glutamine supplementation define the patient groups studied in terms of glutamine deficiency. The original finding by Oudemans-Van Straaten et al. has been confirmed by subsequent studies [35]. It is true that plasma concentration is a poor indicator of intracellular depletion [37], which occurs postoperatively and in critical illness [5,38-40]. This depletion is however not uniform, but different in different cell types [41,42]. A conditional deficiency, as has been suggested, is best characterized by the plasma concentration. During the duration of ICU stay, plasma concentration only changes marginally [5].

To better define the degree of glutamine depletion and to enable a more precise selection of the patients in need of exogenous glutamine supplementation is necessary to be able to design study protocols that will give conclusive evidence [43]. The heterogeneity in patient groups studied, in particular studies of enteral supplementation, may be a major factor behind the inconclusive results [16].

Plasma glutamine concentration is presently the best determiner of glutamine depletion [35,36]. It is usually available and is related to outcome. There is no direct solid knowledge that normalization of plasma glutamine is associated with a better outcome; more than that we know that in the combined group of patients with a normal and/or a normalized plasma glutamine concentration there is a better outcome. More knowledge about the endogenous production of glutamine related to outcome and related to plasma glutamine concentration is needed, in particular when glutamine supplementation is given enterally.

### Possible adverse effects related to glutamine supplementation

The only group of patients in the ICU with supranormal plasma glutamine concentration are patients with acute fulminant liver failure [44]. In contrast patients with chronic liver failure or acute-on-chronic liver failure have low or normal plasma glutamine concentrations and therefore they may be treated as any other patient in the ICU. On the other hand, the patient with acute fulminant liver failure may deserve some caution. Usually the history of disease is short and no malnutrition is at hand, therefore the indication for nutritional intervention is relative. The focus is usually on whether there will be a spontaneous remission or whether an acute liver transplantation will be needed. This decision is usually reached within a few days, so this group of patients does not constitute any major problem. A subgroup--insufficiently studied at present--are patients with acute liver failure after a liver resection with supervening complications. Here, we presently withhold glutamine supplementation waiting for more data.

Head trauma patients and other neurosurgical patients are another group that has been discussed because of the high interstitial concentration of glutamate, which in some observational studies have been associated with worse outcomes. The high interstitial glutamate concentration is mainly reported when microdialysis is done in the so-called penumbra zone, where there is an enhanced cell leakage. Glutamate is an excitatory transmitter and may therefore have effects in itself. In the intact brain, released glutamate is reabsorbed into nerve endings and is reutilized or taken up by astroglia and metabolized into glutamine. Hence, the brain is a net exporter of glutamine. When a supplementation dose of glutamine, on the level of the general recommendation, is given to head trauma patients, no change in the glutamate concentration of the microdialysis fluid can be detected [24]. Furthermore, there is no change in the net balance of glutamine or glutamine across the brain during supplementation [45]. Therefore, fear of an increase of cerebral interstitial glutamine concentration is no reason to abstain from glutamine supplementation. The predicted value of low plasma glutamine concentration or the value of glutamine supplementation on outcome has to our knowledge not been investigated in this particular patient group.

During dialysis or ultrafiltration, small molecules are lost to a higher degree compared with when urine is produced in a healthy kidney. Therefore, the question has been raised whether IV-supplemented glutamine will be lost in the dialysate in particular during continuous renal replacement therapy (CRRT), when the filtration is working around the clock. A recent study showed that all amino acids are lost during CRRT but not glutamine in particular [25]. Nevertheless, when CRRT is necessary the supplementation of glutamine should if anything be a little higher than usual: 0.5 g/kg/day rather than 0.3 g/kg/day.

### Comments on individual studies

Two single-center studies provide the bulk of evidence for the recommendation to give IV glutamine supplementation when TPN is given [14,15]. Both studies are of good quality and involve mainly multiple organ failure patients with a longer ICU stay, but they were performed more than 10 years ago. Both studies show the same pattern, that a tendency toward a reduction in mortality during ICU stay became statistically significant as all-cause 6-month mortality. Both studies were comparatively small (n < 100) and no glucose control was used in the clinical practice at that time. Other studies that are included in the present meta-analyses are of small size or include critically ill patients only as a part of the total patient material [16]. An overview of the cited studies is given in Table 1.

**Table 1:** Overview of cited randomized studies in ICU patients with glutamine supplementation

Study	Year published	n	MC/SC	Patients	EN/PN	Gln adm	Daily gln dose	Length of treatment*	Treatment benefit
Andrews [53]	2011	502	MC	General ICU	EN+PN	iv	10-20 g	5 (1-10)	None
Beale [29]	2008	55	SC	General ICU	EN	enteral	40 g	<10	Morbidity
Conejero [46]	2002	84	MC	General ICU	EN	enteral	35 g	10 (7-25)	Morbidity
Garrel [47]	2003	41	SC	Burns	EN	enteral	26 g	30 ± 17	Mortality
Goeters [14]	2002	95	MC	General ICU	EN+PN	iv	0.2 g/kg	17 ± 11	Mortality
Griffiths [15]	1997	84	SC	General ICU	PN	iv	25 g	6 (2-91)	Mortality
Hall [48]	2003	363	SC	General ICU	EN	enteral	11-27 g	10 (6-16)	None
Houdijk [30]	1998	72	SC	Trauma	EN	enteral	27 g	12 ± 6	Morbidity
Jones [49]	1999	50	SC	General ICU	EN	enteral	18 (11-21) g	11 (3-47)	Morbidity
Wernerman [17]	2011	284	MC	General ICU	EN+PN	iv	0.28 g/kg	12 (4-47)	Mortality
Zhou [50]	2003	40	SC	Burns	EN	enteral	0.35 g/kg	12	Morbidity

MC = multi-center, SC = single-center, EN = enteral nutrition, PN = parenteral nutrition

*Values are medians (ranges) or means ± SD

Studies of enterally administered glutamine supplementation to patients fed by enteral nutrition comprise a much more heterogenic patient group [29,30,46-50]. There are studies of exclusively trauma patients or burn patients, or there are studies that include a broad spectrum of critically ill patients with a wide range of diagnoses and consequently a very variable ICU length of stay. Not surprisingly the meta-analysis shows no mortality advantage but in many individual studies a morbidity advantage [16]. These studies represent an interpretation problem in several respects; in many cases a hypocaloric feeding, uncertainty concerning the utilization of the given supplementation, sometimes a considerable dilution effect from many included short-stayers with a low mortality rate, and a short treatment period [48].

An interesting study with a different concept uses an enteral product with key nutrients, including 30 g of glutamine per day. Some studies employing this product are small in size and mainly focus on safety [51]. One study in multiple organ failure patients, in whom administration of this product was possible, demonstrates an improvement in sequential SOFA scoring [29]. This is the most encouraging finding from enteral administration of glutamine supplementation.

A recent study in Scotland, the SIGNET trial, is the largest (n = 502) study of glutamine supplementation to critically ill patients to date [52,53]. The primary outcome variable was infection rate, and no benefit attributable to the glutamine supplementation was seen. Some concerns about the protocol have been addressed. Glutamine supplementation of 20 g was added into the all-in-one bag containing 2,000 kcal used for parenteral nutrition. Patients were given IV nutrition from this bag whilst the enteral nutrition started in parallel. When more than 50% of the individual caloric target was given as enteral nutrition, the parenteral glutamine containing component was stopped. The result was short-term treatment (mean 5 days), and a low, not well-defined dose of glutamine. In addition, the treatment period was limited to a maximum of 10 days. The obvious limitations from the study make the negative result difficult to interpret.

A Scandinavian glutamine study also has been published recently [17]. In this study, the IV glutamine supplementation was given separate from the nutrition. Supplementation continued as long as patients stayed in the ICU. Patients were fully fed during the entire ICU period, in most cases using a combination of enteral and parenteral nutrition. Primary outcome variable was a difference in SOFA scoring. The study was planned for 1,000 patients, but was interrupted at 410 due to slow recruitment after 4 years. Primary outcome was not conclusive, but as secondary outcome ICU mortality was reduced in the treatment group. This reduction was, however, not sustained as 6-month all-cause mortality. This study is the first to combine enteral and parenteral nutrition. It may be hypothesized from the observation of an ICU mortality effect that continuation of glutamine supplementation also post-ICU may bring an outcome benefit.

In the pipeline there is a large North American-European study combining key nutrients, glutamine, and antioxidants, and combining enteral and parenteral routes for supplementation [54]. Results may be expected 1-2 years from now.

## Conclusions

IV glutamine supplementation is standard care when parenteral nutrition is given in critical illness. For the majority of critical ill patients who are fed enterally, there is insufficient evidence for recommendations presently. Plasma concentration of glutamine is an independent risk factor for mortality in critical illness. So far, low plasma glutamine concentration is the best indicator of glutamine depletion. More knowledge about how the endogenous production of glutamine is regulated is needed to outline better guidelines for glutamine supplementation in the future.

## Competing interests

The author declare no competing economic interests. Being the principal investigator of the Scandinavian Glutamine Trial, there is an academic competing interest, but that study was totally investigator-driven, without any commercial support.
